# Aggressive Cushing’s Disease: Molecular Pathology and Its Therapeutic Approach

**DOI:** 10.3389/fendo.2021.650791

**Published:** 2021-06-16

**Authors:** Masaaki Yamamoto, Takahiro Nakao, Wataru Ogawa, Hidenori Fukuoka

**Affiliations:** ^1^ Division of Diabetes and Endocrinology, Kobe University Hospital, Kobe, Japan; ^2^ School of Medicine, Kobe University, Kobe, Japan; ^3^ Division of Diabetes and Endocrinology, Kobe University Graduate School of Medicine, Kobe, Japan

**Keywords:** Cushing’s disease (CD), aggressiveness/physiology, pathology, medical treatment/surgical treatment, targeted therapy

## Abstract

Cushing’s disease is a syndromic pathological condition caused by adrenocorticotropic hormone (ACTH)-secreting pituitary adenomas (ACTHomas) mediated by hypercortisolemia. It may have a severe clinical course, including infection, psychiatric disorders, hypercoagulability, and metabolic abnormalities, despite the generally small, nonaggressive nature of the tumors. Up to 20% of ACTHomas show aggressive behavior, which is related to poor surgical outcomes, postsurgical recurrence, serious clinical course, and high mortality. Although several gene variants have been identified in both germline and somatic changes in Cushing’s disease, the pathophysiology of aggressive ACTHomas is poorly understood. In this review, we focused on the aggressiveness of ACTHomas, its pathology, the current status of medical therapy, and future prospects. Crooke’s cell adenoma (CCA), Nelson syndrome, and corticotroph pituitary carcinoma are representative refractory pituitary tumors that secrete superphysiological ACTH. Although clinically asymptomatic, silent corticotroph adenoma is an aggressive ACTH-producing pituitary adenoma. In this review, we summarize the current understanding of the pathophysiology of aggressive ACTHomas, including these tumors, from a molecular point of view based on genetic, pathological, and experimental evidence. The treatment of aggressive ACTHomas is clinically challenging and usually resistant to standard treatment, including surgery, radiotherapy, and established medical therapy (e.g., pasireotide and cabergoline). Temozolomide is the most prescribed pharmaceutical treatment for these tumors. Reports have shown that several treatments for patients with refractory ACTHomas include chemotherapy, such as cyclohexyl-chloroethyl-nitrosourea combined with 5-fluorouracil, or targeted therapies against several molecules including vascular endothelial growth factor receptor, cytotoxic T lymphocyte antigen 4, programmed cell death protein 1 (PD-1), and ligand for PD-1. Genetic and experimental evidence indicates that some possible therapeutic candidates are expected, such as epidermal growth factor receptor tyrosine kinase inhibitor, cyclin-dependent kinase inhibitor, and BRAF inhibitor. The development of novel treatment options for aggressive ACTHomas is an emerging task.

## Introduction

Cushing’s disease is a hypercortisolemic state caused by adrenocorticotropic hormone (ACTH)-secreting pituitary adenomas (ACTHomas). Although most ACTHomas can be successfully resected using the transsphenoidal approach, up to 20% of ACTHomas exhibit aggressive behavior, which is defined on the basis of clinical behavior, with a generally invasive, high rate of recurrence, lack of response to optimal standard therapies, or atypical pathological findings including carcinoma like features (1). These result in poor surgical and hormonal outcomes. Crooke’s cell adenomas (CCAs) are one of the well-known aggressive ACTHomas that exhibit characteristic pathological features. ACTHomas can be transformed into an aggressive nature after bilateral adrenalectomy, and such tumors are called Nelson’s syndrome. In pituitary carcinomas, the most aggressive tumoral nature, corticotroph carcinomas followed by or along with PRL-secreting pituitary carcinomas are the most common features. Patients frequently have a clinically serious course due to corticotroph carcinomas, and their management is challenging. In contrast, silent corticotroph adenomas (SCAs) exhibit aggressive tumor behavior, whereas hypercortisolemia is not present. In this review, we summarize our current knowledge of the definition, pathophysiology, and treatment of refractory ACTHomas and provide directions for future research.

## Aggressive ACTHomas

### Crooke’s Cell Adenomas

Crooke’s changes are characterized by large perinuclear cytokeratin filament accumulation in normal corticotrophs due to long-term exposure to endogenous or exogenous glucocorticoid excess, including Cushing’s syndrome. The pathological finding represents an eosinophilic perinuclear hyaline appearance on hematoxylin and eosin staining (2, 3). Crooke’s changes were also discovered within corticotroph adenomas by Kovacs et al. in 1981, called Crooke’s cell adenomas (CCAs) (4). The frequency of Crooke’s changes in ACTHomas varies from 36% to 100% among several reports, and this change is significantly increased in cases with severe hypercortisolism, at least fourfold greater than the upper limit of the normal range of UFC (1, 3, 5). CCA is diagnosed when Crooke’s cells account for more than 50% of the tumor cells (1). These tumors exhibit a high frequency of macroadenoma (77.2%) and more aggressive behavior with invasion to the cavernous sinuses (79.2%) than macro-ACTHomas without Crooke’s change. They can also transform into metastatic pituitary carcinoma, which was previously shown in 7.5% of cases. Some CCAs (24.4%) are clinically silent (6, 7). The recurrence rate of CCAs after surgery is 66% due to the higher frequency of cavernous sinus invasion (8).

### Nelson’s Syndrome

The first case of Nelson’s syndrome was reported in 1958, in a 33-year-old woman who underwent bilateral adrenalectomy due to refractory Cushing’s disease. Three years later, skin hyperpigmentation and visual defects with elevated ACTH levels appeared. These symptoms improved after surgical removal of pituitary tumors, which pathologically exhibited ACTH production (9). Nelson’s syndrome is observed in 8–38% of cases after bilateral adrenalectomy for Cushing’s disease (10, 11).

Accumulating data suggest several risk factors for this syndrome, including a rapid elevation of plasma ACTH after bilateral adrenalectomy (12–14), insufficient steroid replacement therapy (10, 15), residual corticotroph tumor after transsphenoidal surgery (TSS) (16), younger age (17), and histopathological characteristics of corticotroph tumor specimens. Elevation of plasma ACTH levels of more than 100 pg/mL in the first year after bilateral adrenalectomy is associated with the development of Nelson’s syndrome (18).

From a histological point of view, there is no difference between the tumors of Nelson’s syndrome and those of Cushing’s disease. Despite the low expression of Ki-67 (usually less than 3%), tumor behavior is aggressive and invasive. The most common clinical manifestation of Nelson’s syndrome is dark skin hyperpigmentation, with markedly elevated plasma ACTH levels. Bitemporal hemianopia and progressive visual loss caused by aggressive tumors are clinically important issues to be addressed. Therefore, regular follow-up should be monitored using MRI.

### Corticotroph Carcinoma

Pituitary carcinomas are currently defined as pituitary tumors with craniospinal dissemination or metastasis to other types of tissues (19). Pituitary carcinomas occupy only 0.1–0.2% of pituitary neoplasms derived from the anterior pituitary (19). Epidemiologically, there is no gender difference in the prevalence of pituitary carcinomas (20). Metastasis is commonly identified in the central nervous system, followed by the liver, bones, and lungs (20). The specific symptoms of pituitary carcinoma are absent and depend on the region of metastasis, such as hearing loss, ataxia, or motor impairment (21). While CT and MRI are most often utilized to identify the metastatic region, ^18^F-FDG, ^111^In-labeled octreotide, and ^68^Ga-DOTANOC in scintigraphy are also useful according to recent case reports (21–23).

There are no pathological criteria to distinguish aggressive adenomas from carcinomas. The following morphological features are not useful in predicting malignant transformation of pituitary adenomas: hypercellularity, nuclear and cellular pleomorphism, increased mitotic activity, necrosis, and dural and/or bony invasion, which are generally associated with malignancy (24, 25).

In endocrinological manifestation, 85–90% of carcinomas express pituitary hormone, and 15–20% of them are clinically nonfunctioning. Prolactin or ACTH-secreting carcinomas are the most frequent, followed by growth hormones and other rare hormones (23, 26–28). In a literature review, corticotroph carcinomas were shown to be the most common (34.7%) among pituitary carcinomas, followed by prolactin-secreting (23.6%) and null cells (15.3%) (20). Corticotroph carcinoma is usually developed from a group that exhibits aggressive phenotypes, such as invasive, rapid growth, and prone to recurrence. One case series of 31 patients with CCAs exhibited more than 80% with single or multiple recurrences, and two patients developed corticotroph carcinoma (6). Nelson’s syndrome occurring after bilateral adrenalectomy has also potentially progressed to carcinomas (21, 29). Furthermore, malignant transformation of SCAs has been reported in rare case reports (30).

Regarding prognosis, one case series of 15 patients with pituitary carcinoma reported that 66% of the patients died within 1 year and 20% were alive at the last follow-up 9–18 months after diagnosis (28). A recent review showed that 34 of 62 patients (55%) died within approximately 10 months after the diagnosis of pituitary carcinoma (31–33).

### Silent Corticotroph Adenomas (SCAs)

SCAs are defined as ACTH-expressing pituitary tumors that lack both the clinical symptoms of Cushing’s syndrome and evidence of autonomous cortisol secretion, which is diagnosed with nonfunctioning pituitary adenomas (34–36). The clinical importance of differentiating SCAs from other nonfunctioning pituitary adenomas is due to their aggressive nature (37). The prevalence of SCAs ranges from 3% to 6% in all pituitary adenomas and less than 40% in corticotroph adenomas (36–39).

Patients with SCAs are younger and predominantly females and have a higher prevalence of giant adenomas and an association with cavernous sinus invasion than other nonfunctioning pituitary adenomas, such as silent gonadotroph adenomas (SGA) and null cell adenomas (40, 41). In imaging studies of nonfunctioning pituitary adenomas, cystic or hemorrhagic changes on MRI T2WI are observed in SCAs (42). Regardless of tumor size, multiple microcystic changes are more frequently observed within SCAs and are highly specific to SCAs. These multiple microcysts have been correlated with pseudopapillary features of SCA pathological findings (42). According to the WHO classification, SCAs are classified into two subtypes: type 1 (densely granulated) and type 2 (sparsely granulated) (24, 34, 41). Type 1 SCAs show strong ACTH immunoreactivity similar to typical ACTHomas, while type 2 SCAs exhibit weak and focal expression (43). The lack of galectin-3 expression in corticotroph adenomas can be pathologically diagnostic of SCAs rather than functioning corticotroph adenomas (44). In type 2 SCAs, the expression levels of fibroblast growth factor receptor-4, matrix metalloproteinase-1, and β1-integrin which associates with tumor aggressiveness, are higher than that in type 1 SCAs (45), suggesting different tumor pathologies, however it remains unclear whether these two subtypes indeed influence tumor behavior.

The underlying mechanism of the discrepancy between elevated ACTH levels and normocortisolemia remains unclear in these patients. Various hypotheses have been reported to date. First, SCAs are driven from an intermediate lobe, which, in turn, demonstrates a low ACTH secretory capacity (30, 46). However, this concept was not established in a subsequent study (47). As a second hypothesis, SCAs secrete predominantly unprocessed high-molecular-weight ACTH (also known as big ACTH), which causes competition with mature ACTH at the receptor binding level (48). Another mechanism was suggested to be an increased intracellular degradation of ACTH, which resulted in insufficient ACTH exocytosis from the cell membrane (49). As the most widely accepted concept, the expression levels of prohormone convertase (PC), which is a critical enzyme in POMC posttranslational processes, determine the characteristic difference between SCAs and Cushing’s disease. POMC is cleaved by PC1/3 and PC2 into biologically active ACTH and alpha-MSH, respectively (50). Several reports have demonstrated that SCAs exhibit decreased protein expression levels of PC1/3 concomitant with PC1/3 mRNA downregulation compared to typical corticotroph adenomas (35, 51).

In a certain portion of corticotroph adenomas, bidirectional transformation of the tumor phenotype between SCA and Cushing’s disease has been reported (52) in 3.9% of cases, with a transformation period ranging from 1 to 7 years (52). Interestingly, an altered expression level of PC1/3 has been observed with the lapse of time in identical pituitary adenomas. The clinical phenotype correlates with *PC1/3* mRNA or protein levels in corticotroph adenomas (43), suggesting that PC1/3 expression levels play an important role in determining the characteristics of these tumor phenotypes.

## Molecular Pathology

### Typical ACTHomas and Their Aggressiveness

The most common genetic cause of ACTHomas is the somatic *ubiquitin-specific protease 8* (*USP8*) variant within the 14-3-3 binding motif, which accounts for approximately 20–60% of these tumors (53, 54). The underlying mechanisms of *USP8* variants are thought to be mediated by increasing the deubiquitylation activity of this enzyme, leading to epidermal growth factor receptor (EGFR) overexpression (53). EGFR overexpression in corticotrophs has been proven to be a pathogenesis of ACTHomas due to its enhanced proliferation and ACTH hypersecretion (55, 56). In *USP8* wild-type ACTHomas, the p.Met415 variant within the catalytic domain of *USP48* has been identified (57). However, these genetic variants are found in small tumors and are not associated with tumor aggressiveness in ACTHomas. The *BRAF* p.V600E variant, which is also found in other cancers, has been identified in ACTH-secreting macroadenomas (57). However, tumor behavior remains unclear due to its low frequency (58).

Next-generation sequencing of *USP8* wild-type ACTHomas has revealed *TP53* pathogenic variants that are associated with larger and invasive tumors including tumors from patients with Nelson’s syndrome or pituitary carcinomas (58, 59). *TP53* variants with wild-type *USP8* are associated with chromosome instability, aneuploidy, and tumor aggressiveness (60). Another possible gene associated with tumor aggressiveness is *CABLES1*, a major glucocorticoid-dependent cell cycle regulator in corticotrophs (61). Recently, mutations in the *ATRX* gene, which is one of the driver mutations in neuroendocrine tumors and regulates chromatin remodeling and telomere maintenance, have been shown to be associated with aggressive pituitary adenomas, especially in ACTHomas (62). *ATRX* gene mutations are associated with a lack of ATRX expression in tumor specimens. Loss of function somatic mutations have been found in the *CABLES1* gene in children or young adult patients with aggressive corticotroph adenomas (63).

As germline mutations, *MEN1, PRKAR1A, CDKN1B*, and *AIP* genes should be considered as a young age-onset genetic syndrome phenotype (64). In aggressive pediatric Cushing’s syndrome, the *DICER1* gene has been reportedly identified to have a causal role in ACTH-producing pituitary blastoma caused by DICER1 syndrome (65–67) (**Table 1**). Since DICER1 is an enzyme required for the cleavage of a precursor into mature microRNA, noncoding RNA, including microRNA, are associated with the pathogenesis of pituitary ACTH-producing tumors and their aggressiveness.

**Table 1:** Causative Genes in Cushing’s disease and its association with aggressiveness.

Gene	SIG	Frequency	Aggressive
*USP8*	Both S>G	20-60% in CD	No
*USP48*	S	13.3% in CD	No
*MEN1*	Both G>S	1% in genetic syndrome	No
*CDKN1B*	G	Rare - 2.6% in pituitary adenomas	No
*PRKAR1A*	G	Very Rare	No
*AIP*	Both G>S	5% of FIPA	Possibly No
*BRAF*	S	Rare	Possibly Yes
*CABLES1*	Both	2.2% in CD	Yes
*DICER1*	G	Very rare	Yes
*TP53*	S	12.5% in CD	Yes
*ATRX*	S	28% in PC	Yes
		13% in APT	

S, somatic variants; G, germline variants; CD, Cushing’s disease; PC, pituitary carcinomas; APT, aggressive pituitary tumors.

### Crooke’s Cell Adenomas (CCAs)

The genetic causes of CCAs have not been elucidated. In histopathologic findings, the Ki-67 score has limitations in predicting tumor proliferation and aggressiveness (6, 19). Rather than the Ki-67 labeling index, miR-106b–25 and its host gene *MCM7*, a member of the minichromosome maintenance complex (MCM) family of proteins, have been shown to be novel markers that correlate with tumor recurrence and progression in invasive ACTH-producing pituitary adenomas, including CCAs (68, 69).

### Nelson’s Syndrome

The underlying mechanisms of pituitary tumorigenesis and autonomous ACTH secretion in Nelson’s syndrome are not fully understood. Corticotropin-releasing hormone (CRH) hyperactivity induced by rapid cortisol reduction is thought to be one of the pathogenesis of its marked ACTH elevation and aggressive tumor enlargement. These tumors were originally derived from monoclonal cells (70, 71). Essential transcription factors (e.g., Ptx1, Tpit, NeuroD, Nur77) in corticotroph cells and *POMC* gene posttranscriptional processes are properly conserved, which results in a mature POMC product (72–74). Regarding the molecular function of corticotroph tumors, CRHR1 and AVPR1b receptors on the tumor exhibit good responsiveness to their ligands (72–75). Intriguingly, loss of heterozygosity of the glucocorticoid receptor (GR) gene has been reported in patients with Nelson’s syndrome, while GR expression of the corticotroph tumor in Nelson’s syndrome is conserved, similar to that in Cushing’s disease (76, 77). In some tumors in Nelson’s syndrome, TP53 loss of function has been identified after radiation therapy (78). The primary management mode (pituitary surgery and radiotherapy (RT) followed by adrenalectomy) of Cushing’s disease before the diagnosis of Nelson’s syndrome has been reported as the highest risk and a predictor of tumor progression (79).

### Pituitary Carcinomas

The pathogenesis of pituitary carcinomas is not fully understood due to its low frequency. However, *TP53* variants or *ATRX* variants have been shown in some corticotroph pituitary carcinomas (59, 62). Lynch syndrome, which is caused by *MSH2* gene mutation complicated with pituitary carcinoma, has been reported as a case report, showing an association between this tumor-prone syndrome and pituitary tumors (80). However, further investigations need to clarify the tumor transformation’s underlying mechanism to the malignant behavior in pituitary adenomas.

### Silent Corticotroph Adenomas

The transformation from functioning ACTHomas into SCAs is very rare (3.9%) (52). From an autopsy pathological study of the human pituitary gland, it has been suggested that SCA originates from pars intermedia POMC-positive cells, while ACTHomas originate from the anterior lobe (46). This hypothesis was confirmed by an animal study using tamoxifen-inducible Pax7^CreERp/WT^ Rb^flox/flox^ mice, which revealed that Rb loss in the Pax7-expressing pituitary intermediate lobe results in cell proliferation leading to tumorigenesis expressing POMC without circulating ACTH elevation (81). Regarding the genetic cause of SCAs, candidate genes have not yet been clarified, and the *USP8* mutation commonly found in functioning and silent corticotroph adenomas (30, 82). Immunohistochemical analysis of SCA has demonstrated that lower expression of some proteins implicated in tumor progression and metastasis, such as galectin-3, a beta-galactoside-binding protein, and KLK10, belonging to the kallikrein family, in SCA than functioning ACTHomas may be one mechanism of its aggressiveness (30). Lower expression of CDKN2A with upregulated cyclin D1 in SCA than functioning ACTHomas has been shown to be another reason for its aggressive behavior (83). Recently, gene and protein expression comparison analysis between SCAs and functioning ACTHomas has been performed using both RNA-seq and mass spectrometry-based proteomics technology, revealing the downregulation of the gene related to protein processing in the endoplasmic reticulum (ER) pathway and upregulation of *PCSK1N*, an inhibitor of PC1/3 coding *PCSK1* gene. These results suggest a reason for the lack of active ACTH secretion from these tumors. Moreover, the extracellular matrix (ECM) protein cluster is downregulated in SCAs compared to functioning ACTHomas, suggesting that this is associated with their invasive behavior (84).

## Treatment

Surgery is the first-line treatment to control tumor volume for refractory corticotroph tumors, even though the postoperative recurrence rate remains high (8, 85). Radiosurgery is an important option for treating postoperative residual tumor or recurrence and progression of tumors. Stereotactic radiosurgery is especially superior to radiosurgery in terms of a lower incidence of adverse events and earlier remission (86, 87). In aggressive ACTHomas, medical therapy is required, followed by surgery and radiosurgery in most cases. Although hypercortisolemia can be controlled by adrenal or GR-targeted drugs, the effect of targeted therapy on ACTHoma remains a challenge. Medical treatment can be initiated immediately after the diagnosis until hypercortisolemia is collected, including the perioperative period.

### Surgery

Surgical treatment remains the first-line treatment choice even for aggressive ACTHomas by a skilled neurosurgeon with extensive experience in pituitary surgery (88). Endoscopic or microscopic TSS can be performed according to the neurosurgeon’s preference (89). Preoperative medical treatment to improve hypercortisolemia is recommended, mainly using steroidogenesis inhibitors, including metyrapone, ketoconazole, and osilodrostat with or without hydrocortisone replacement (90–92). Since a higher rate of morbidity, including poorly controlled diabetes mellitus, hypokalemia, venous thromboembolism, gastrointestinal hemorrhage, and osteoporosis, has been complicated in patients with refractory Cushing’s disease, several pharmaceutical treatments such as insulin, mineral corticoid antagonists, anticoagulants, proton pump inhibitors, and anti-osteoporotic agents are required during the perioperative period (91, 93). In a recent systematic review, the complete surgical remission and recurrence rates of macro-tumors in primary surgery were 68% (95% confidence interval [CI]; 60–76) and 30% (95% CI; 18–43), and those in revision surgery are 49% (95% CI; 23–75) and 45% (95% CI; 0–98), respectively (94). If the tumor extends into the suprasellar region, a transcranial approach may be needed (88). In a literature review of initial surgery for ACTHomas, a tumor with Knosp grades 3–4 have been identified in 12–20% of all tumors, and 12 of 36 patients (33.3%) have achieved the remission criteria (95), indicating that further treatment option is emergently required for these tumors. In Nelson’s syndrome, pituitary surgery is the first-line treatment; however, the complete remission rate depends on whether the pituitary tumor extends to the extrasellar region, similar to an ordinal pituitary tumor (96).

### Radiotherapy (RT)

Stereotactic RT (SRT), including the Gamma Knife™ (GK), Cyberknife™, and proton-beam RT, has become the mainstream rather than the conventional fractionated RT (CRT) and could be a second treatment option for aggressive Cushing’s disease if residual or recurrent tumors are visible on MRI despite TSS (97). Because of the recent development of drug therapy, the choice of second-line therapy needs to be individualized according to tumor progression speed by MRI, pathological findings, and patient background. In a systematic review from 2000 to 2017, the tumor was controlled in 95% of cases (83.3–100%) with a median follow-up of 56 months (2–17 years). Hormonal control has achieved 54–68% in SRT with a follow-up of 5–10 years, while the definition of biochemical remission is not unified. However, the recurrence rate of RT is 20–32% with a median time of 25.5–37 months (range 6–60) after an initial remission. Adverse radiation effects for patients with Cushing’s disease including hypopituitarism [12.3–52% (median 22.6%)], visual toxicity (0–39%), and cranial nerve neuropathy (0–5.5%) have reported, while secondary brain tumors have not occurred yet. The median time to hormonal normalization is 12–25 months (98–102). In aggressive Cushing tumors, the mean time of hormonal control may have taken a longer period than that in those with nonaggressive ones (33.0 ± 5.0 *vs.* 23.5 ± 6.3 months) (101). In patients with CCAs, SRT has shown to be as effective as ACTHomas without Crooke’s hyaline changes (87).

### Medical Therapy

There are three therapeutic targets for drug therapy in patients with Cushing’s disease: pituitary directed therapies, adrenal directed therapies, and cortisol-target tissues. In aggressive Cushing’s disease, which is usually accompanied by remarkable hypercortisolemia, adrenal gland-targeted steroidogenesis inhibitors, including ketoconazole, metyrapone, and etomidate, can be the first choice for acute phase intervention or preoperative treatment. Further medical treatment might be required in the chronic phase, or if there are residual tumors that oversecrete ACTH after the operation of the tumors. Pituitary gland-targeted drugs, such as second-generation somatostatin receptor ligands (SRLs), pasireotide, cabergoline is a dopamine receptor agonist, could be the next treatment choice with or without steroidogenesis inhibitors.

**Figure 1:**
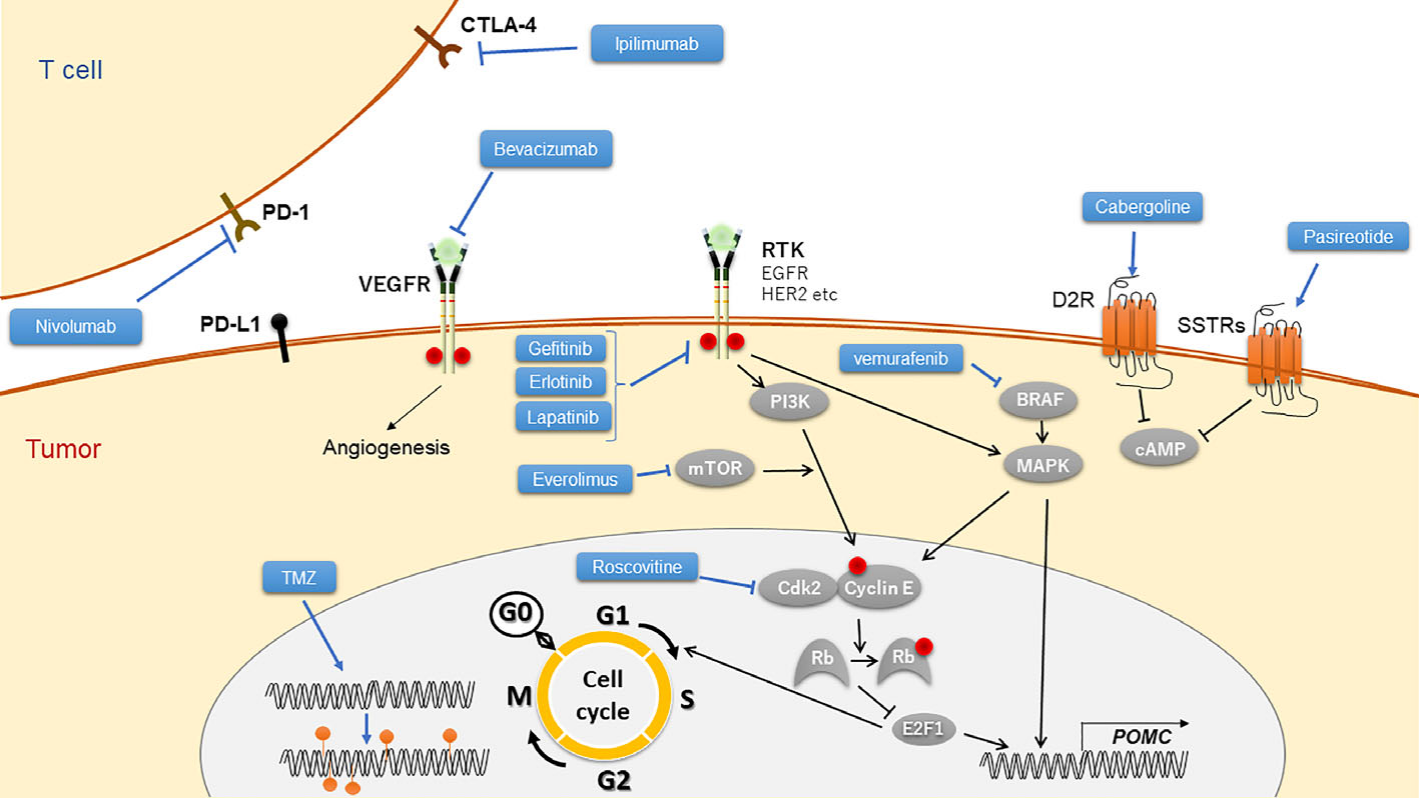
Targeted drugs to Cushing’s disease and their mechanistic scheme. TMZ, temozolomide; RTK, receptor of tyrosine kinase.

Our manuscript mainly focused on pituitary gland-targeted therapies including currently approved and further developing drugs (**Figure 1**), besides described adrenal gland-targeted drugs and peripheral GR blockers.

### Somatostatin Receptor Ligands

First-generation SRLs, octreotide and/or lanreotide, which mainly target SSTR2, are not effective in most ACTHomas because of their low expression of membrane SSTR2. Pasireotide, a second-generation SRL that targets SSTR1, 2, 3, and 5 with the highest affinity for SSTR5, has been approved as a promising drug for the treatment of ACTHomas (103, 104). In a recent meta-analysis, pasireotide was shown to be effective in normalizing cortisol in 41.1% (95% CI, 32.7–49.8) of patients (105).

In ACTH-secreting macroadenomas, ACTH reduction and tumor shrinkage have been reported by initial treatment of pasireotide (106). Furthermore, the effectiveness of rapid ACTH and cortisol suppression as preoperative treatment has been shown in several ACTH-secreting macroadenoma cases (107). Conversely, escape from ACTH reduction, or even a paradoxical rise of ACTH has been reported (108), indicating further investigation to clarify the effect of this drug on aggressive ACTHomas.

The effect of pasireotide on CCAs is still under debate and requires further studies to clarify the efficacy of such challenging aggressive tumors. In Nelson’s syndrome, a sufficient effect on ACTH reduction and tumor shrinkage has been reported in a case report of pasireotide (109). A multicenter trial of pasireotide treatment for Nelson’s syndrome has been reported (110). In this trial, patients were treated with subcutaneous (s.c.) pasireotide twice daily for 1 month (n=8), followed by treatment with monthly pasireotide LAR for 6 months (n=5). ACTH reduction showed a complete response (CR) in five out of eight patients and partial response (PR) in two out of eight patients by s.c. pasireotide and further exhibited a CR in three out of five patients and PR in one out of five patients treated with pasireotide LAR. However, tumor shrinkage was not observed with hyperglycemia in six patients.

SCAs also exhibit a higher expression of SSTR2 and SSTR5 compared to null cell adenomas and SGAs by immunohistochemical analysis (40, 111). SSTR3 is also abundantly expressed in SCAs (40). Although the efficacy of somatostatin analogs for SCAs has not yet been established, pasireotide LAR (PASSILCORT; ClinicalTrial.gov identifier, NCT02749227) is under a phase II randomized clinical trial for residual or recurrent SCAs.

### Dopamine Receptor Agonist

Since dopamine 2 receptor (D2R) is frequently expressed on ACTHomas, the dopamine receptor agonist cabergoline has shown to be an effective drug in approximately 20–30% of patients with Cushing’s disease (112–114). In a recent multicenter study, the efficacy of cabergoline in hormone reduction did not differ between microadenomas and macroadenomas (112). The effect of cabergoline on CCAs remains unclear (8). Although some case reports show that Nelson’s syndrome has been successfully treated with cabergoline (115, 116), the efficacy of cabergoline for such aggressive tumors is limited (79). In SCAs, a case report revealed that cabergoline has been shown to induce tumor shrinkage in a patient with SCA, in which D2R expression has been proved by *in situ* hybridization (117). However, SCAs have been reported to exhibit lower D2R mRNA levels than ACTH-negative nonfunctioning adenomas (118).

### Temozolomide

Temozolomide is the drug with the most developed evidence for the treatment of aggressive pituitary adenomas and pituitary carcinomas. Although insurance is not covered in most countries, temozolomide is a promising therapeutic choice for refractory hormone-secreting and non-secreting pituitary tumors, including Cushing’s disease.

This drug was initially used in the treatment of glioblastoma multiforme (GBM) because of its significant clinical benefits. It is an alkylating agent that methylates specific guanine residue, leading to DNA damages by triggering tumor apoptosis. However, the existence of O6-methylguanine-DNA methyltransferase (MGMT), a DNA repair enzyme that can remove the methyl from the O6-methylguanine, works in contrast with temozolomide. Therefore, high MGMT expression levels in GBM tumors are related to drug resistance (119).

The first two cases of pituitary carcinomas were reported in 2006, who were successfully treated with temozolomide after initial therapy including surgery, dopamine agonists, somatostatin analogs, radiation, and chemotherapy (120). Following this report, more than 150 cases with pituitary carcinomas or aggressive pituitary adenomas have been treated with temozolomide, demonstrating a 69% (33–86%) response rate, which is defined as either a complete remission (CR), partial response (PR), or stable disease (SD). Additionally, 42% (29–69%) of significant tumor volume reduction has been shown to be either a CR or PR (88).

The reduction in tumor size occurred within 1–6 months after initiation of temozolomide therapy. Noting a report from a European cohort, after 3 (6) cycles of temozolomide, 23% (59%) achieved maximal radiological response, indicating that approximately 40% of patients experienced maximal radiological tumor response after 6 months (88, 121). Moreover, a German survey also reported that 52% of corticotroph tumors showed regression, 21% stability, and 26% progression at the end of temozolomide treatment. After a median follow-up of the 32-month radiological evaluation, disease stabilization in 37% and progression in 63% of patients were observed (121). These results are consistent with previous findings in Italy and France (122).

Although randomized prospective trials, or head-to-head studies of temozolomide compared to placebo or other treatment options, have not been performed yet, temozolomide can be a potential recommended therapy of choice for aggressive pituitary adenomas and pituitary carcinomas.

In aggressive pituitary tumors, Cushing’s disease is the most common type, with 45% of adenomas and 47% of carcinomas (88). Generally, functioning tumors, especially prolactinomas and corticotroph adenomas, have been reported to have a better response to temozolomide than nonfunctioning tumors. The response rate of temozolomide in corticotroph tumors is estimated to be 56%, compared to 44% in prolactinomas, 38% in somatotroph tumors, and only 22% in nonfunctioning tumors (89), indicating that aggressive corticotroph adenoma and corticotroph carcinomas are good candidates for temozolomide treatment. In fact, five cases of CCAs, which showed lower MGMT expression than noninvasive ACTHomas, treated with temozolomide, were shown to have a partial or complete reduction of tumor size in all cases. Plasma ACTH levels in these cases have also been suppressed, except for one case in which laboratory data were not documented (123). In addition, a German survey reported that ACTH decreased from 42 (9–794) pmol/L at baseline to a minimum of 23 (10–276) pmol/L after a median of 6 (3–10) months on temozolomide and then increased to 182 (12–671) pmol/L at the end of temozolomide treatment (122).

In children, temozolomide treatment for aggressive pituitary adenoma and carcinoma is quite rare, which leads to insufficient treatment data. In limited cases of *DICER1* mutations with ACTH-secreting pituitary tumors, the effect of temozolomide has not been clearly shown (65, 124). Although there is no established course of treatment because of the paucity of data, the ESE guidelines suggest that temozolomide might be beneficial in adults (88). The common adverse events observed were similar to those observed in adults, including diarrhea, constipation, nausea, thrombocytopenia, headaches, syncope, and somnolence (36, 125–127).

In SCAs, temozolomide is considered a possible chemotherapeutic agent because of its low expression of MGMT (36, 126, 127). Several case reports of SCAs treated with temozolomide have been reported. Ceccato et al. reported two SCA patients treated with temozolomide: one associated with pasireotide treatment showed stable disease (SD) with 6% volume reduction and the other showed partial response (PR) with 49% volume reduction (128). A systematic review and meta-analysis reported that the recurrence rate of SCAs with a mean follow-up of <5 years or >5 years was 25% and 31%, respectively, and there was no significant difference in the recurrence rates between SCAs and other nonfunctioning pituitary adenomas (129). However, it should be noted that there are many unresolved points because of the rarity of SCAs.

When pituitary tumors show metastatic spread or are refractory to multiple treatments, temozolomide could be the last resort and salvage therapy. However, some recent studies have suggested that early use of temozolomide in these patients could result in a better outcome (88, 121). In this regard, high-grade tumors on MRI, such as invasiveness and increasing tumor size, and pathological findings, including high MIB1-labeling index, could be a sign of temozolomide initiation after surgery under RT (130, 131). However, patients administered with temozolomide in the early stage are relatively rare. Further clinical investigations are needed to determine whether early administration of temozolomide in patients with aggressive or metastatic pituitary tumors is associated with better outcomes.

Tumors resistant to TMZ chemotherapy have been shown in a certain number of refractory pituitary tumors (130). Thus, a predictive marker for resistance to temozolomide needs to be identified. As shown in GBM, low MGMT expression, a beneficial predictor of the response to temozolomide in glioblastoma (119), has been mostly associated with a positive response to temozolomide in pituitary tumors. However, some discrepancies, such as a high MGMT with a lack of response and no response despite low MGMT expression, have also been reported in pituitary tumors (88). Furthermore, no statistical association between MGMT expression levels and resistance to temozolomide has been shown (121, 122), indicating the limitation of MGMT as a predictive marker in these tumors. In addition to MGMT, several DNA mismatch repair (MMR) pathway proteins have been proposed, including MLH1, MSH2, MSH6, and PMS2, which recognize adducts including O6-methylguanin and remove them, leading to cell death (132). Therefore, the expression levels of MMR proteins may be critical to the cytotoxic effects of temozolomide (88). In fact, MSH6 immunopositivity has been associated with responsiveness to temozolomide in malignant pituitary neoplasms (133). Further analysis of the relationship between MMR pathway protein expression levels and temozolomide responsiveness is required (132). Overall, the expression of DNA repair proteins, including MGMT, may be associated with resistance to temozolomide treatment but is still controversial.

### Chemotherapy

There are no established chemotherapy protocols for pituitary carcinomas. Mono- or combination therapy using chemotherapy, including capecitabine, carboplatin, etoposide, cisplatin, doxorubicin, 5-fluorouracil, tamoxifen, cyclophosphamide, lomustine, procarbazine, vincristine, oxaliplatin, dacarbazine, methotrexate, bleomycin, and cyclohexyl-chloroethyl-nitrosourea, has been used for several pituitary carcinoma cases, with some PR (20, 134–136).

### Immune Therapy

As a successful immunotherapeutic strategy, immune checkpoint inhibitors (ICIs) for several cancers, has recently been developed. These targets include cytotoxic T lymphocyte antigen 4 (CTLA-4) and programmed cell death protein 1 (PD-1), located in T cells, and ligand for PD-1 (PD-L1), located in tumor cells. Ipilimumab, the first developed ICIs targeting CTLA-4; nivolumab, pembrolizumab, and cemiplimab targeting PD-1; and atezolizumab, avelumab, and durvalumab targeting PD-L1, have been approved and applied for the treatment of several cancers. For pituitary tumors, the first case treated with ICIs has been reported to have ACTH-secreting pituitary carcinoma exhibiting liver metastasis (137). In this patient, initial treatment, including TSSs, fractionated RT and pasireotide, and cabergoline, was performed followed by TMZ and a combination of TMZ and capecitabine. Since the tumor volume and hormonal hypersecretion were not controlled despite these treatments, the combination of ipilimumab (3 mg/kg every 3 weeks) and nivolumab (1 mg/kg every 3 weeks) was initiated, leading to regression of both sellar tumors and metastatic liver tumors (59% and 92%, respectively) with a 90% reduction in ACTH levels. In this report, genetic analysis of primary and metastatic tumors revealed several pathogenic somatic gene hypermutations possibly induced by medical therapy such as TMZ, which can be neoantigens for the targets of ICIs. Following this report, successful treatment of a second corticotroph carcinoma case derived from Nelson’s syndrome, with a combination of ipilimumab (3 mg/kg) and pembrolizumab (1 mg/kg) every 3 weeks, leading to stable disease, has been reported (138). Immunotherapy could be the next possible therapeutic candidate for aggressive ACTHomas.

### Possible Targeted Therapy

Drug repositioning from several targeted therapies for cancers, including neuroendocrine tumors to aggressive pituitary tumors, has been investigated using receptors for tyrosine kinases, including EGFR, human epidermal growth factor receptor 2 (HER2), vascular endothelial growth factor receptor (VEGFR), intracellular signal transduction pathway proteins such as the mammalian target of rapamycin (mTOR), BRAF, and nuclear proteins such as cyclin-dependent kinase (CDK) (136, 139).

In ACTHomas, EGFR has been shown to be a tumorigenic factor, especially in *USP8* variants (53, 56, 140). Although ACTHomas with *USP8* mutations have been shown to be small and nonaggressive, EGFR overexpression in ACTHomas has been reported to be associated with aggressive ACTHomas *via* the activated MAPK pathway (141). Since EGFR can induce experimental corticotroph tumor proliferation both *in vitro* and *in vivo*, its tyrosine kinase inhibitor (TKI) gefitinib has been shown to reduce serum corticosterone levels with shrinking pituitary tumors of corticotroph-specific EGFR overexpressing mice, an animal model of Cushing’s disease (55, 56). EGFR TKI erlotinib and dual EGFR and HER2 TKI lapatinib have been used to treat aggressive pituitary tumors as a third-line treatment, revealing poor outcomes (142). Recently, lapatinib treatment for aggressive PRLomas has been reported to have a partial effect on tumor shrinkage and hormonal reduction (143). Further investigation of EGFR targets is required for aggressive corticotroph tumors.

VEGF inhibitors, including TKIs for VEGFR2, and monoclonal neutralizing antibodies against VEGF-A have been used for several vascular-rich cancers, including neuroendocrine neoplasms. The rationale of these drugs is to suppress angiogenesis, leading to the suppression of tumor growth and induction of shrinkage. Bevacizumab, a humanized monoclonal antibody for VEGF-A, has been used to treat SCA, leading to stable disease for at least 26 months (144). Eight years of PFS with RT, TMZ, and bevacizumab has been reported (145). Five more cases of ACTHoma treated with bevacizumab have been reported, showing some effectiveness (146) since VEGF can also modulate the tumor microenvironment, their inhibition can act as an antitumor immunity (147).

In pituitary adenomas, mTOR expression has been shown to be higher than those in a normal pituitary gland and is elevated in invasive tumors (148, 149), suggesting a potential therapeutic target of mTOR for aggressive ACTHomas. Everolimus, an mTOR inhibitor, has been approved for several cancer treatments, including neuroendocrine neoplasms (150). Everolimus has been reported as an effective therapy for *STK11* mutated refractory ACTHoma with clinical improvement and stable disease for at least 6 months (151). In contrast, one case of corticotroph carcinoma was treated with everolimus with octreotide, exhibiting resistance in both tumoral growth and hormone secretion. According to the microarray investigation, *regulatory associated protein of mTOR* (*RAPTOR*) mRNA expression was low, suggesting the cause of everolimus resistance in corticotroph carcinoma (152).

In ACTHomas with *BRAF*, V600E mutation, a rare variant, has been shown to be a good candidate for the treatment with the BRAF inhibitor vemurafenib (57).

From the Cushing’s disease model of corticotroph-specific pituitary tumor transforming gene (PTTG) transgenic zebrafish, drug screening has been performed, identifying that the CDK2/cyclin E inhibitor, R-roscovitine, could be a potential drug for human ACTHomas (153, 154).

### Adrenal Gland-Targeted Drugs

#### Steroidogenesis Inhibitors

Adrenal steroidogenesis inhibitors block cortisol synthesis by inhibiting various enzymes in steroidogenesis pathway while they have no evidence in corticotroph tumor shrinkage.

Ketoconazole is known as an anti-fungal biotics, which can inhibit cholesterol side-chain cleavage enzyme such as 17α-hydroxylase and 17, 20-lyase and 11β-hydroxylase (155). Ketoconazole which has numerous evidence in treating hypercortisolemia due to Cushing’s syndrome, exhibited high remission rate from 45 to 93% (91). Liver enzyme elevation is one of the most common side effects, which was observed in 13.5% of patients. As other adverse events, gastrointestinal disturbances and male hypogonadism should be considered (91).

Mitotane is currently approved for treating adrenocortical carcinoma, also widely used for Cushing’s syndrome, by inhibiting not only steroidogenesis but also inducing cell death of adrenocortical cells. According to a recent meta-analysis, mitotane exhibited high remission rate in treating Cushing’s syndrome (105), While dyslipidemia, gastrointestinal disturbances and neurological disorders are frequently observed adverse events, mitotane-induced adrenal insufficiency requires special caution, which demand more glucocorticoid dose than physiological setting.

Metyrapone is widely used as a steroidogenesis inhibitor for Cushing’s syndrome even though it has been still in off-label use in US. Metyrapone inhibits 11β-hydroxylase and converting from 11-deoxycortisol to cortisol, results in reducing cortisol level. Metyrapone showed a revised estimated average remission rate of 75.9% (105) The frequently reported adverse events were hirsutism in women, dizziness, arthralgias, gastrointestinal disturbances, adrenal insufficiency, hypokalemia and peripheral edema (156).

### Novel Steroidogenesis Inhibitors

#### Levoketoconazole

Levoketoconazole which is an enantiomer of ketoconazole, was developed for achievement of better efficacy and safety. Levoketoconazole inhibits 21-hydroxylase, 17 alpha-hydroxylase, and 11 beta-hydroxylase steroidogenesis enzymes, resulted in exhibiting higher potency than ketoconazole (157). In phase 3 clinical trial, treated 81% patients with levoketoconazole achieved normalization of UFC level. While most of adverse events such as nausea and headache were acceptable, 13% of patients was obliged to discontinue the drug due to serious adverse events such as abnormal liver functio, prolonged QT interval, and adrenal insufficiency (158).

#### Osilodrostat

Osilodrostat is novel 11 beta-hydroxylase inhibitor that blocks the conversion from deoxycortisol to cortisol, which has similar action mechanism with metyrapone. Osilodrostat exhibited 3-fold higher affinity to 11 beta-hydroxylase and longer half-life than metyrapone. In please II clinical trial, osilodrostat treatment reduced UFC in 78.9% of patients at week 22 (159). Adverse events were very similar with those of other steroidogenesis inhibitors, including nausea, diarrhea, asthenia, adrenal insufficiency, and hirsutism in female (160).

#### Glucocorticoid Receptor-Directed Drugs

Mifepristone is officially approved non-selective GR antagonist for treating Cushing’s syndrome. The data from a multicenter, open-label, prospective clinical trial showed the improvement of clinical features associated with hypercortisolemia, psychiatric symptoms and glucose intolerance (161). On the other hand, specific inhibition of GR action causes hyperaldosteronism-like phenotype due to cortisol binding to MR, such as hypertension and hypokalemia. Apart from that, various adverse events also have been reported as follows: nausea, fatigue, and endometrial thickening in women (162).

## Conclusions

In this review article, we introduced several aggressive types of ACTHomas, including CCAs, Nelson’s syndrome, and SCAs. The pathogenesis and treatment of these tumors have been introduced. Although numbers of genetic variants and mutations are implicated in ACTHomas, their mechanistic link to the aggressiveness and, more importantly, to therapeutical targeting are yet to be established. Future targeted drugs and immunotherapy are shown with their potential evidence. Further analysis and investigation are urgently required for this clinically serious disease.

## Author Contributions

MY wrote the sections “Aggressive ACT Homas” and “Molecular Pathology”. TN wrote the section “Temozolomide”. The rest was written by HF. HF and WO planned and edited this review. All authors contributed to the article and approved the submitted version.

## Funding

Grant-in-Aid for Scientific Research from the Japanese Ministry of Education, Culture, Sports, Science and Technology 15K09432, 19K09003.

## Conflict of Interest

The authors declare that the research was conducted in the absence of any commercial or financial relationships that could be construed as a potential conflict of interest.
